# Defects of the Lunacy Law

**Published:** 1924-04

**Authors:** 


THE
### HOSPITAL and HEALTH REVIEW

### DEFECTS OF THE LUNACY LAW.

MORE than sixty years have elapsed since Charles Reade wrote "Hard Cash," and during the whole of those two generations there has been an uneasy suspicion in the public mind that all is not well with our lunacy law and our lunatic asylums. The recent remarkable case in which Mr. W. S. Harnett was awarded £25,000 damages against two doctors for wrongful detention has awakened these suspicions to active existence, and the Government are committed to what the Prime Minister describes as "a serious inquiry" into the lunacy laws and their administration. Upon the case which has produced this decision we have no intention of entering. An appeal from the verdict and judgment will be heard shortly, and, pending the re-hearing, newspapers are rightly precluded from discussing the merits. There are, however, certain considerations arising out of the case which are of the utmost moment to every man and woman walking the earth in freedom and to some at least of the unhappy persons who are within the walls of public and private asylums. The partitions which divide a sound from an unsound mind are so thin that, until our knowledge of mental science is vastly increased, cases must constantly arise which hover on the border line, and perhaps are sometimes on one side of it and sometimes on the other. Mr. Harnett was under confinement for nine years, and the jury found that he was sane all the time. Whether the jury or the doctors were more likely to be right is a point not meet for present discussion. We merely state what the jury found.

In the course of the case Mr. Justice Lush expressed his astonishment at two facts which are certainly not generally known. One is that if an inmate of an asylum, sane or insane, manages to escape and to evade recapture for a fortnight he cannot be taken back. The process of certification must be gone through afresh before he can again lawfully be confined. There is no logic in such a provision of the statute law, and it is obvious that an escaped and uncertificated lunatic roaming about the world is an acute public danger. The other—and much more amazing—fact is that "when a man is shut up in an asylum, although he may be a perfectly sane man, he is never allowed to know why he is shut up." The words are those of the judge, who may well have been "amazed" by such an astonishing discovery. The theory, no doubt, is that an insane person would be incapable of understanding the nature of the certificate if it were shown to him ; but what of the possibility of his sanity ? A man who is charged with a crime is entitled to receive, and does in fact receive, the fullest information of the charge before it is heard ; the alleged lunatic often does not so much as realise that his sanity is suspect. No one who has any real knowledge of the subject would subscribe to the grotesque notion that our asylums are full of sane people—those who take up that attitude would seem to be themselves on the wrong side of the bars. But, the Harnett case apart, cases do unquestionably occur in which persons of oscillating mind do not, for one reason or another, obtain the benefit of the doubt or in which the signs of recovery are insufficiently closely observed. The "mad doctor" is as honest and conscientious as any other kind of doctor, or as those who are too ready to condemn him for his supposed heartlessness or carelessness, but he follows an extremely difficult and hazardous profession in which it is peculiarly easy to make a disastrous mistake.

The course of the inquiry promised by the Government, when at last the way is clear for it, will be followed with real anxiety by the public, and it is highly desirable that it should result in recommendations which will set uneasiness at rest and make mistakes, rare as they are, less easy. It may be that the process of certification needs to be surrounded by greater safeguards and that there should be more thorough and more frequent examinations into the condition of patients who are not obviously incurable. This might be accomplished by giving the lunacy justice who is called upon to sign the certificate the help of a mental specialist independent of the medical certifying authority, and the review of the detention order within a very short period. It goes without saying that the person alleged to be insane, or some one representing him, should have the absolute right to see the certificate. There ought clearly, in every case, to be a preliminary stage before definite and final certification—a kind of certificate *nisi*—pending the reasonable certainty which would justify a decree absolute. But even then there should be careful periodical reviews. It is imperative that we should get rid of the suspicion that sane people are in confinement, consorting with those who really have lost their reason. Nothing could be more perilous to the mind consciously sane than daily intercourse with the mad, and terrible as is the plight of the prisoner convicted yet innocent, that of the sane person who is officially a lunatic is infinitely worse. The very urgency and ceaselessness of his representations may seem uncommonly like monomania, and may tell more powerfully against him than in his favour. Some of the acutest and most highly trained minds in the ranks of medicine are devoted to the study of the diseased brain, and every year, thanks to their investigations, we learn more of the conditions which make for mental abnormality. It is not their fault, but the fault of the law, that men and women may be incarcerated without knowing why, or that other things may happen which assuredly ought to be impossible. Reforms which will place the lunacy laws upon a more rational foundation and protect both patient and alienist will be welcomed as readily by them as by outside critics.

